# A developing *Setaria viridis* internode: an experimental system for the study of biomass generation in a C_4_ model species

**DOI:** 10.1186/s13068-016-0457-6

**Published:** 2016-02-24

**Authors:** Antony P. Martin, William M. Palmer, Christopher Brown, Christin Abel, John E. Lunn, Robert T. Furbank, Christopher P. L. Grof

**Affiliations:** School of Environmental and Life Sciences, University of Newcastle, University Drive, Callaghan, NSW 2308 Australia; Department of Metabolic Networks, Max Planck Institute of Molecular Plant Physiology, Am Mühlenberg 1, 14476 Potsdam, Germany; ARC Centre of Excellence for Translational Photosynthesis, Research School of Biology, Australian National University, Canberra, ACT 2601 Australia; CSIRO Agriculture Flagship High Resolution Plant Phenomics Centre, GPO Box 1600, Canberra, ACT 2601 Australia

**Keywords:** *Setaria viridis*, RNAseq, Stem, Bioenergy, Cell wall, Sugar, C_4_ grass

## Abstract

**Background:**

Recently, there has been interest in establishing a monocot C_4_ model species with a small genome, short lifecycle, and capacity for genetic transformation. *Setaria viridis* has been adopted to fill this role, since reports of Agrobacterium-mediated transformation in 2010, and sequencing of its genome in 2012. To date, *S. viridis* has primarily been used to further our understanding of C_4_ photosynthesis, but is also an ideal system for the study of biomass crops, which are almost exclusively C_4_ panicoid grasses. Biogenesis of stem tissue, its cell wall composition, and soluble sugar content are important determinants of bioenergy crop yields. Here we show that a developing *S. viridis* internode is a valuable experimental system for gene discovery in relation to these important bioenergy feedstock traits.

**Results:**

The rate of maximal stem biomass accumulation in *S. viridis* A10 under long day growth was at the half-head emergence developmental stage. At this stage of development, internode 5 (of 7) was found to be rapidly expanding with an active meristem, a zone of cell expansion (primary cell walls), a transitional zone where cell expansion ceased and secondary cell wall deposition was initiated, and a mature zone that was actively accumulating soluble sugars. A simple method for identifying these zones was established allowing rapid dissection and snap-freezing for RNAseq analysis. A transcriptome profile was generated for each zone showing a transition from cell division and nucleic acid synthesis/processing in the meristem, to metabolism, energy synthesis, and primary cell wall synthesis in the cell expansion zone, to secondary cell wall synthesis in the transitional zone, to sugar transport, and photosynthesis in the mature zone.

**Conclusion:**

The identification of these zones has provided a valuable experimental system for investigating key bioenergy traits, including meristematic activity, cell wall biosynthesis, and soluble sugar accumulation, in a C4 panicoid grass that has genetic resources, a short life cycle, and small stature allowing controlled experimental conditions in growth cabinets. Here we have presented a comprehensive map of gene expression and metabolites in this experimental system to facilitate gene discovery and controlled hypothesis testing for bioenergy research in *S. viridis*.

**Electronic supplementary material:**

The online version of this article (doi:10.1186/s13068-016-0457-6) contains supplementary material, which is available to authorized users.

## Background

*Setaria viridis*, the wild relative of foxtail millet (*Setaria italica*), has recently been adopted as a model species for the study of C_4_ panicoid grasses by the photosynthesis research community [1–6]. The support of the research community for this model is evidenced by the first *Setaria* conference held in Beijing in 2014 [7]. Reported uses of *Setaria viridis* to date have focused on the collection of natural diversity panels [8, 9], the development of mapping populations [10], transformation [11–14] and crossing [15] techniques, and generation of molecular resources such as transformation vectors [12] and notably a genome sequence [16]. Most research using these resources has been directed towards the study of C_4_ photosynthesis and associated leaf development and cellular differentiation [17]; however, *S. viridis* is also a valuable model species for the study of biomass crops, which are almost exclusively C_4_ panicoid grasses (e.g. giant miscanthus (*Miscanthus x giganteus*), switchgrass (*Panicum virgatum*), sugarcane (*Saccharum officinarum*), maize (*Zea mays*), and sorghum (*Sorghum bicolor*)) sharing a highly similar lineage to *Setaria*. Whilst photosynthesis is an important determinant of crop biomass yield, knowledge of the factors controlling biogenesis of stem sink tissue, its cell wall composition, and soluble sugar content are crucial for the improvement of forage and bioenergy crop quality and yields.

The main agricultural products of forage and bioenergy crops are soluble sugars and the cell wall fractions of culms, which are used as a source of feed or fuel. To maximise yields, larger culm volumes with high sugar content and easily hydrolysed cell wall polymers are required. The cell wall composition of *S. viridis* has been shown to be consistent with that of major bioenergy crops with equivalent conversion rates of cellulose to fermentable sugars [18]. No study of soluble sugar levels in *S. viridis* culms has been published; however, Brix concentrations of up to ten can be achieved (Martin et al. unpublished observations), which is equivalent to that of mid-range sweet sorghum [19] and sugarcane [20] crops. *Setaria viridis* culms therefore possess attributes that suggest they would be a valuable C_4_ model system for the study of not only cell wall synthesis and stem development but also sugar accumulation in C_4_ panicoid grass culms.

A grass culm consists of multiple repeating phytomeric units, comprised of a node and an internode. Stalks develop and elongate acropetally, whilst individual internodes develop and elongate basipetally with a sigmoidal elongation pattern [21]. At the midway stage in its development, an internode will contain an active intercalary meristem at its base, acropetally followed by a cell expansion zone, before transitioning into mature cells towards the top. Based on early physiological studies of oat culm development [22] and extensive literature in sugarcane and sweet sorghums, which show the accumulation of sugar in mature internodes [23–25], the following picture emerges for a developing internode in panicoid grass species. The meristem contains actively dividing cells undergoing mitosis and cell differentiation processes, and the expansion zone contains cells that are actively expanding under turgor whilst synthesising primary cell walls and cell membranes. As the cells mature, secondary cell wall deposition terminates cell expansion and the cells realise their capacity to import and store sugar.

Here we characterise a developing *S. viridis* internode that contains an active intercalary meristem, rapidly expanding cells with primary cell walls, a transitional zone where secondary cell wall synthesis is initiated, followed by a mature zone where sugar import and storage are occurring. RNA sequencing of tissues from each of these developmental zones provides a framework for gene discovery. Ultimately, genetic manipulation of genes discovered here has the potential to alter sink size by affecting cell division and expansion in the meristematic and cell expansion zones, and to influence the molecular composition of cell wall polymers and soluble sugars by affecting metabolic pathways in the transitional and maturation zones. Whilst there have been other transcript studies conducted in grass stems at various stages of development, including young and mature maize internodes [26], and in bamboo [27], this is the first study to characterise and utilise the developmental zones within an elongating internode, and the first whole RNAseq transcriptome dataset for *S. viridis* stem tissue. This experimental system and transcriptomic resource in the C_4_ model species, *S. viridis*, will facilitate gene discovery with respect to grass culm development and allow controlled experimental exploration of traits relevant to bioenergy crops such as culm sink size, cell wall composition, and soluble sugar content. The genetic and physiological similarities between *Setaria viridis* and key bioenergy crops will increase the translatability of results into forage and bioenergy crop improvement programmes.

## Results

**Fig. 1:**
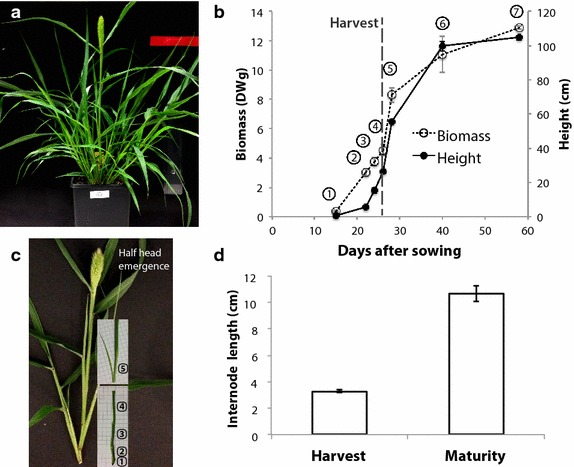
Selection of a rapidly elongating internode. **a** *S. viridis* A10 grown under controlled conditions at half-head emergence developmental stage. **b** Main stalk height and sucrose concentration measured at seven developmental stages: 6-leaf (*1*), 9-leaf (*2*), booting (*3*), half-head emergence (*4*), anthesis (*5*), milky dough (*6*), and maturity/hard dough (*7*). The harvested stage (half-head emergence) is indicated with a *dashed line*. **c** The main stalk was stripped of its leaf sheath with internodes labelled 1–5 (counting only internodes ≥5 mm in length). **d** Internode 5 length measured at harvest and at maturity. All values are mean ± SE of five biological replicates

To identify a rapidly expanding internode, *S. viridis* plants were grown under highly controlled conditions and seven developmental stages were examined. The fourth developmental stage, half-head emergence, was defined as the point when half of the seed head had emerged from the leaf sheath (Fig. 1a). This was chosen as the developmental stage with the most rapid rate of stem growth, which also corresponded with the most rapid rate of sucrose accumulation (Fig. 1b). At this developmental stage, the leaf sheath was carefully stripped to expose the stem and its constituent internodes. Internode five, counted from the bottom (considering only internodes ≥5 mm in length), or, interchangeably, the second internode from the top (not including the flag internode), was selected as a rapidly elongating internode containing well-defined elongation and mature zones (Fig. 1c). To ensure this internode was rapidly elongating, the same internode was also measured in mature plants and was shown to increase from an average length of 3.8 cm at harvest (half-head emergence) to 10.2 cm at maturity (Fig. 1d).

To define the meristematic, cell expansion, transitional, and mature zones of internode five, nuclei density and cell size were examined using fluorescence microscopy. It was also essential to develop a method for visually identifying these zones of internode five so that harvest for RNAseq analysis could be achieved rapidly, without any need for microscopy, and therefore without excessive tissue damage.

**Fig. 2:**
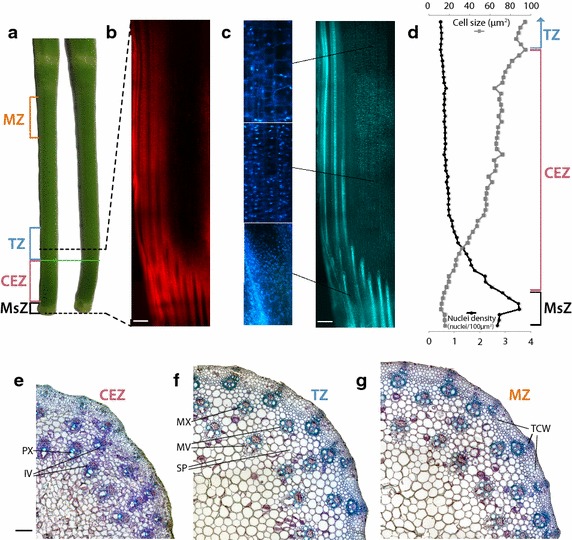
Defining meristematic (MsZ), cell expansion (CEZ), transition (TZ), and cell maturation (MZ) zones of internode 5. **a** Internode 5 harvested at the half-head emergence developmental stage with its leaf sheath stripped, alongside an equivalent internode 5 where the lower, flexible zone has been bent to display the differentiation between the upper rigid zone and the lower flexible zone. The *green line* indicates the interface between the flexible and rigid zones of the internode. The harvested meristematic (MsZ—*black*), cell expansion (CEZ—*pink*), transitional (TZ—*blue*), and mature (MZ—*orange*) zones are indicated. **b** and **c** Longitudinal, vibratome cut, 50-μm thick section of the lower region of the internode (indicated by *dashed black lines*), stained with DAPI and viewed under UV illumination with **b**, the *red* chlorophyll emissions isolated, and **c**, the *blue* DAPI emissions isolated. Enlarged regions are offset in **c**, and *white scale bars* are 50 μm. **d** Nuclei density and cell size measured using image J were plotted at intervals along the lower region of the internode. **e**, **f** and **g**, Microtome cut 2-μm-thick cross-sections from e, the cell expansion, **f**, transitional, and **g**, mature zones, stained with Toluidine blue, counter stained with Lugol’s iodine and imaged using brightfield illumination. Protoxylem (PX), immature vascular bundles (IV), metaxylem (MX), mature vascular bundles (MV), storage parenchyma (SP), and thickened cell walls (TCW) are labelled. *Black scale bars* in **e**, **f**, and **g** are 100 μm

Internodes were photographed (Fig. 2a), whilst intact and then embedded in agarose and sectioned longitudinally. Nuclei were stained with DAPI, illuminated with UV light and viewed using a long-pass DAPI filter that allowed red chlorophyll fluorescence (Fig. 2b) and blue DAPI fluorescence of nuclei (Fig. 2c) to be visualised simultaneously. Processing DAPI images allowed nuclei density (a proxy for the number of cells) and also cell size to be plotted along the length of the internode (Fig. 2d). A spike in nuclei density mapped to a well-defined white band at the base of the internode, and this was defined as the meristematic zone (MsZ) (Fig. 2a) allowing for rapid identification of this zone. Directly above the meristematic zone, cell size increased and nuclei density decreased, both asymptotically. It was observed that this cell expansion zone was dark green in colour, which was also indicated by the increased red chlorophyll fluorescence observed in longitudinal sections of this region (Fig. 2b). The base of the internode was flexible (Fig. 2a), likely due to the lack of lignified secondary cell walls. The point where the internode became rigid corresponded with the end of the chlorophyll-enriched zone and the approach of nuclei density and cell size towards their asymptote. Both the end of the chlorophyll-enriched zone and the point where the internode became rigid were therefore used as markers to dissect the cell expansion zone (CEZ) (Fig. 2a) of the internode without the need to stain or section the tissue. The region of the internode directly above this point, cut to ~85 % the length of the CEZ, was dissected and defined as the transition zone (TZ) (Fig. 2a). A zone immediately below the node above internode five, cut at the same length as the CEZ, was dissected as the mature zone (MZ) (Fig. 2a).

Using brightfield microscopy, it was evident that there were many different cell types within these zones. The cell expansion zone contained immature vascular bundles with protoxylem and very little cell wall thickenings in any cell types (Fig. 2e). The transition zone contained fully developed vascular bundles with fully developed xylem, but only modest cell wall thickenings (Fig. 2f). The mature zone contained fully developed vascular bundles with fully developed xylem and more cell wall thickening than the transitional zone (Fig. 2g). Based on evidence from these images, the dissected zones contained a variety of cell types, but were sufficiently enriched in expanding, transitioning, and mature tissues to reveal differences in metabolite levels and gene expression between each developmental stage.

**Fig. 3:**
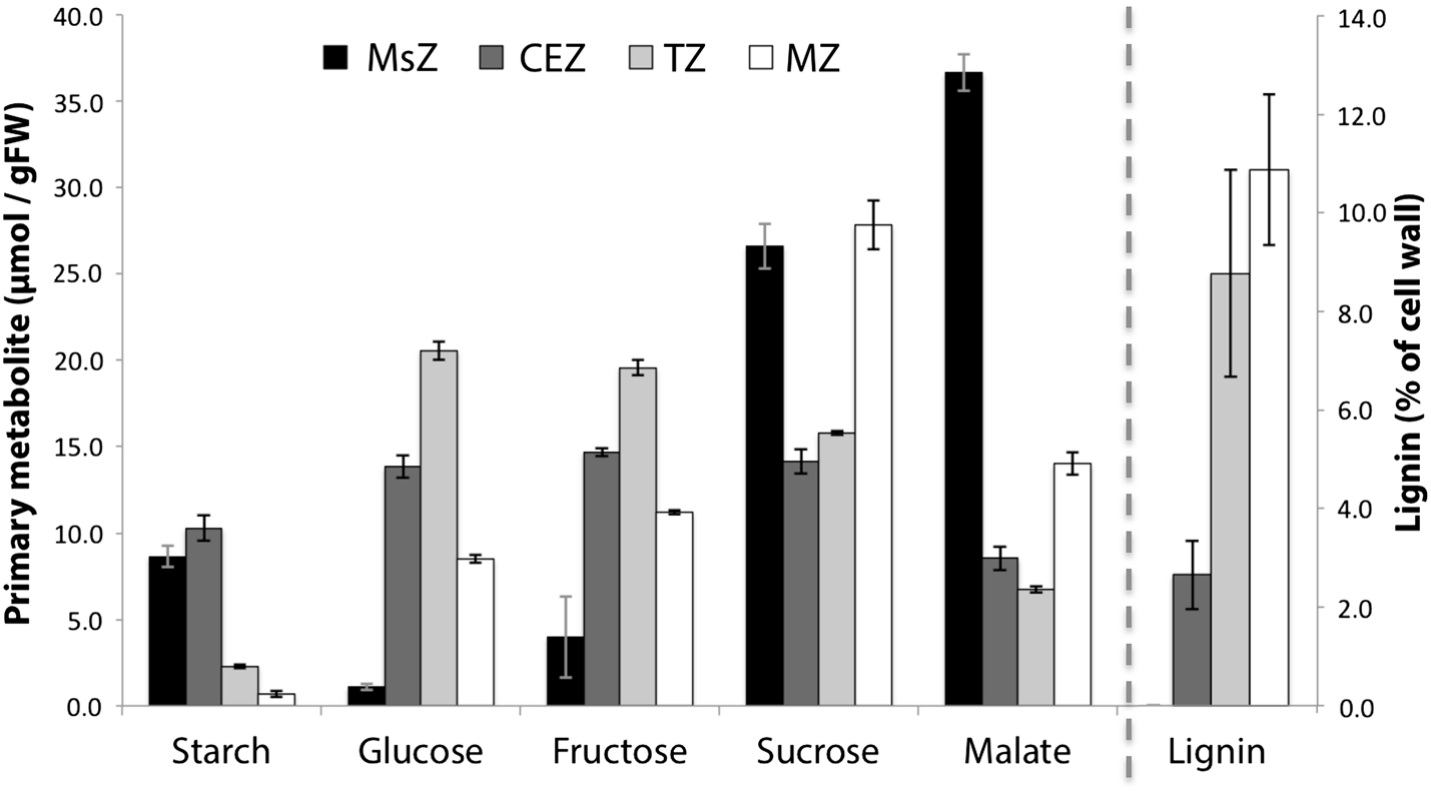
Primary metabolites and cell wall lignin in the developing internode. Starch (measured as molecules of released glucose), glucose, fructose, sucrose, and malate were assayed enzymatically in extracts from the meristem, cell expansion, transition, and mature zones of the fifth internode. Lignin was measured using the acetyl bromide method. All values are mean ± SE of four biological replicates of pooled material from ten plants

Primary metabolites were measured in the four zones of the developing internode to establish carbon pools (Fig. 3). Starch was found only in the developmentally young meristematic and cell expansion zones. The ratio of sucrose to hexoses was high in the meristematic zone, reduced in the growing cell expansion and transitional zones, and increased in the mature zone, which was likely at the beginning of a sugar storage phase as sugar levels rise towards the maximal concentration observed in stage 6 (Fig. 1b). High levels of the soluble C_4_ acid malate were also measured and correlated strongly with sucrose levels. The role of malate in this developing C_4_ internode is unclear; however, it may be involved in a functional C_4_ pathway, used as a pH regulator, a storage molecule in vacuoles, or an osmolyte for turgor regulation [28].

The lignin content of cell walls was also measured in the cell expansion, transitional, and mature internode zones as an indicator of the presence of primary or secondary cell walls (Fig. 3). Very low levels of lignin were observed in the cell expansion zone; however, the cell walls rapidly became lignified in the transitional zone, and continued to accumulate lignin up to the mature zone (Fig. 3). This suggested that the cell expansion zone contained primary cell walls, whereas the transitional zone was rapidly synthesising secondary cell walls, and that this synthesis was maintained, up to higher levels in the mature zone where it has, likely, completed lignification.

**Fig. 4:**
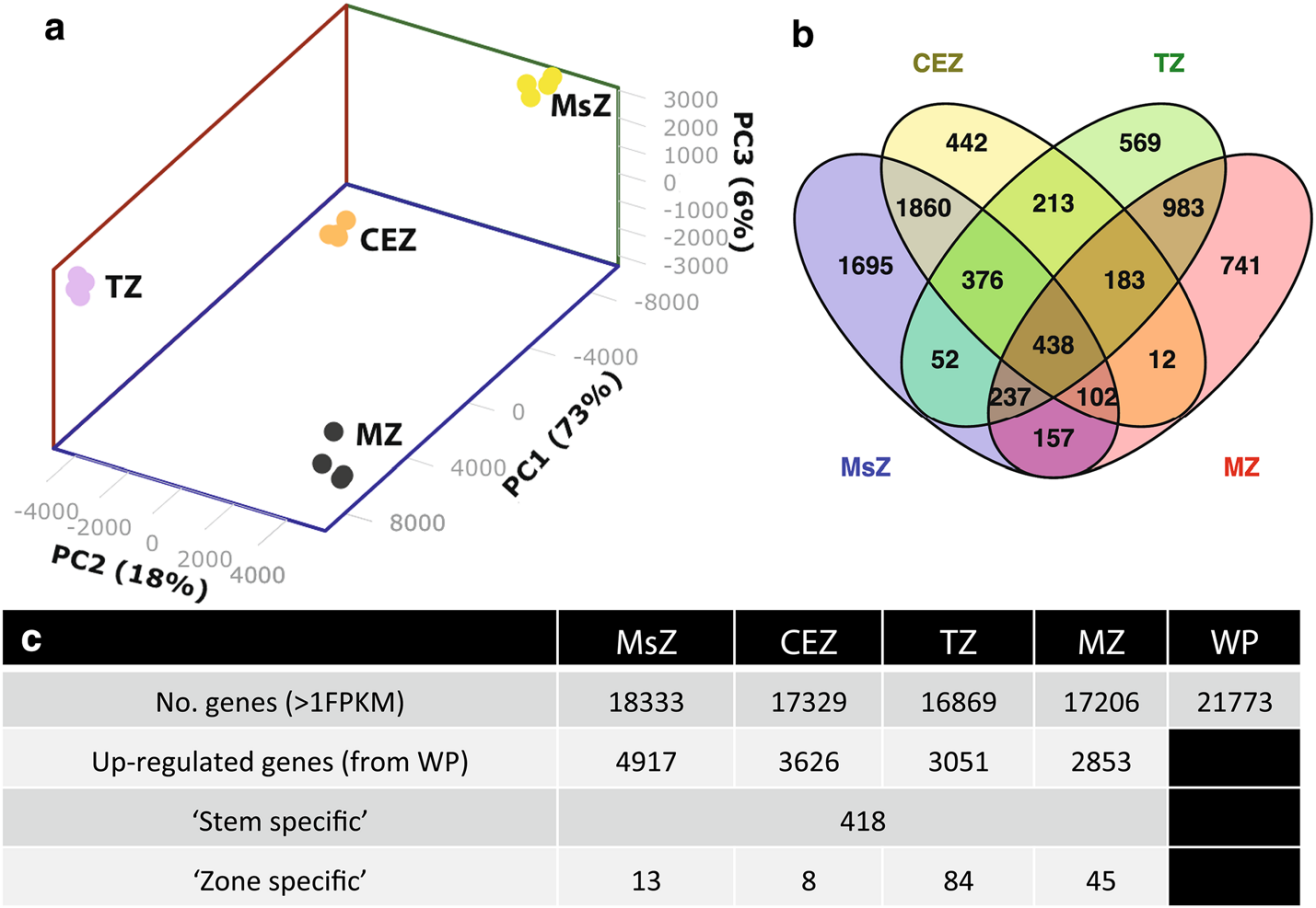
Internode zones have unique RNAseq profiles and contain stem- and zone-specific genes. **a** Principal component analysis of FPKM values obtained from RNA sequencing of the MsZ, CEZ, TZ, and MZ of internode 5. **b** *Venn diagram* of genes up-regulated greater than a log_2_ fold change of one against the background levels in the whole plant (WP) transcriptome. **c** *Table* showing the number of genes expressed >1 FPKM, number of genes up-regulated against the WP transcriptome with >1 log_2_ fold, number of identified ‘stem-specific’ genes with >2.5 log_2_ fold change against the WP background and >80 FPKM in any internode zone, and the number of ‘zone-specific’ genes with, in addition to being ‘stem-specific’, >2 log_2_ fold change against all other internodal zones (with some exceptions, see Additional file 2)

Once the developmental status of the four internode zones was established, RNA was extracted, sequenced, mapped (see Additional file 1 for mapping details), and expression values calculated. Principal component analysis (PCA) of global gene expression confirmed that each internodal zone had a unique gene expression profile with the meristematic and cell expansion zones clustering separately from the transitional and mature zones (Fig. 4a). Very little variation between biological replicates was also observed confirming the reproducibility of the harvesting technique.

Comparing log_2_ fold-change expression of each gene to that obtained from a ‘whole plant’ background transcriptome [3], lists of genes that were up-regulated (>1 log_2_ fold) in each zone were generated and displayed as a Venn diagram (Fig. 4b). This also revealed a dichotomy between the two younger meristematic and cell expansion zones, and the two older transitional and mature zones. Thousand eight hundred and sixty genes were up-regulated in common in MsZ/CEZ and 983 in TZ/MZ. In comparison, only 52 MsZ/TZ, 157 MsZ/MZ, 213 CEZ/TZ, and 12 CEZ/MZ genes were up-regulated in common (Fig. 4b). The number of up-regulated genes and the number expressed above a threshold of one fragments per kilobase per million reads (FPKM) also showed dichotomy with more being found in younger than older tissues (Fig. 4c). This suggests that greater biological complexity is required for meristematic activity and cell expansion than for the established biological processes of mature tissues including secondary cell wall synthesis and carbon storage.

Using criteria of gene expression above a level of 80 FPKM (chosen by visually examining read coverage of genes with ~80 FPKM), and a log_2_ fold change >2.5 against the whole plant (WP) background transcriptome, 418 ‘stem-enriched’, 13 ‘MsZ-enriched’, 8 ‘CEZ-enriched’, 84 ‘TZ-enriched’, and 45 ‘MZ-enriched’ genes were identified which can be used to identify promoter sequences for the construction of tissue-specific, and likely cell-specific, expression vectors (‘enriched’ genes are marked in Additional file 2).

**Fig. 5:**
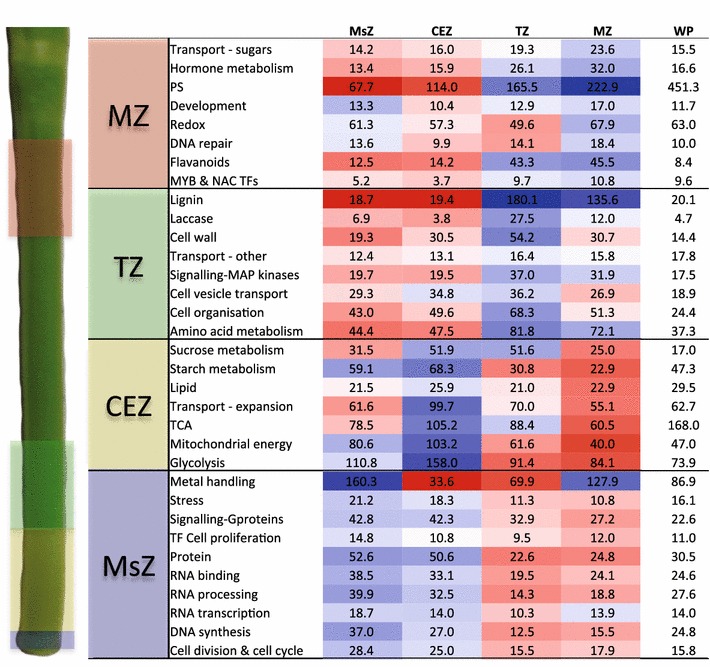
Meristematic, cell expansion, transition, and mature zones are enriched in unique gene families. Average expression of selected gene families, based on the Mapman annotations, arranged into categories that are enriched in the meristematic (MsZ), cell expansion (CEZ), transition (TZ), and mature (MZ) zones of the internode. Mean expression is tabulated for MsZ, CEZ, TZ, MZ, and whole plant (WP) and the heat map is generated from the log_2_ fold change across the four internode zones. *Blue* indicates higher expression, whilst *red* indicates lower expression, and values are given as mean FPKM for each gene category

To further characterise the segregation of biological processes within each zone of the developing internode, genes were categorised based on the most recent Mapman ontology, which was updated for this analysis (Additional file 3). The meristematic zone was enriched in processes related to cell division and cell cycle including DNA, RNA, and protein synthesis/regulation, confirming that this zone is actively undergoing cell division. The cell expansion zone was enriched in metabolism, energy production, and lipid biosynthesis, which are required to drive cell expansion. The transitional zone was enriched in secondary cell wall biosynthesis-related gene categories including lignin biosynthesis, laccases, cell vesicle transport, MYB, and NAC transcription factors (which are maintained in the mature zone), and amino acid metabolism. The mature zone was enriched in sugar transporters, photosynthesis (which begins to operate, however, not to the levels observed in the whole plant), and flavonoid biosynthesis (Fig. 5). These transcript profiles confirm that the transcriptional machinery required for stem-specific intercalary meristem cell division and differentiation is active in the meristematic zone; energy-driven cell expansion is active in the cell expansion zone; secondary cell wall synthesis, including transcriptional switches, is active in the transitional zone; and sugar unloading, storage, and possibly synthesis (photosynthesis) is active in the mature zone.

**Fig. 6:**
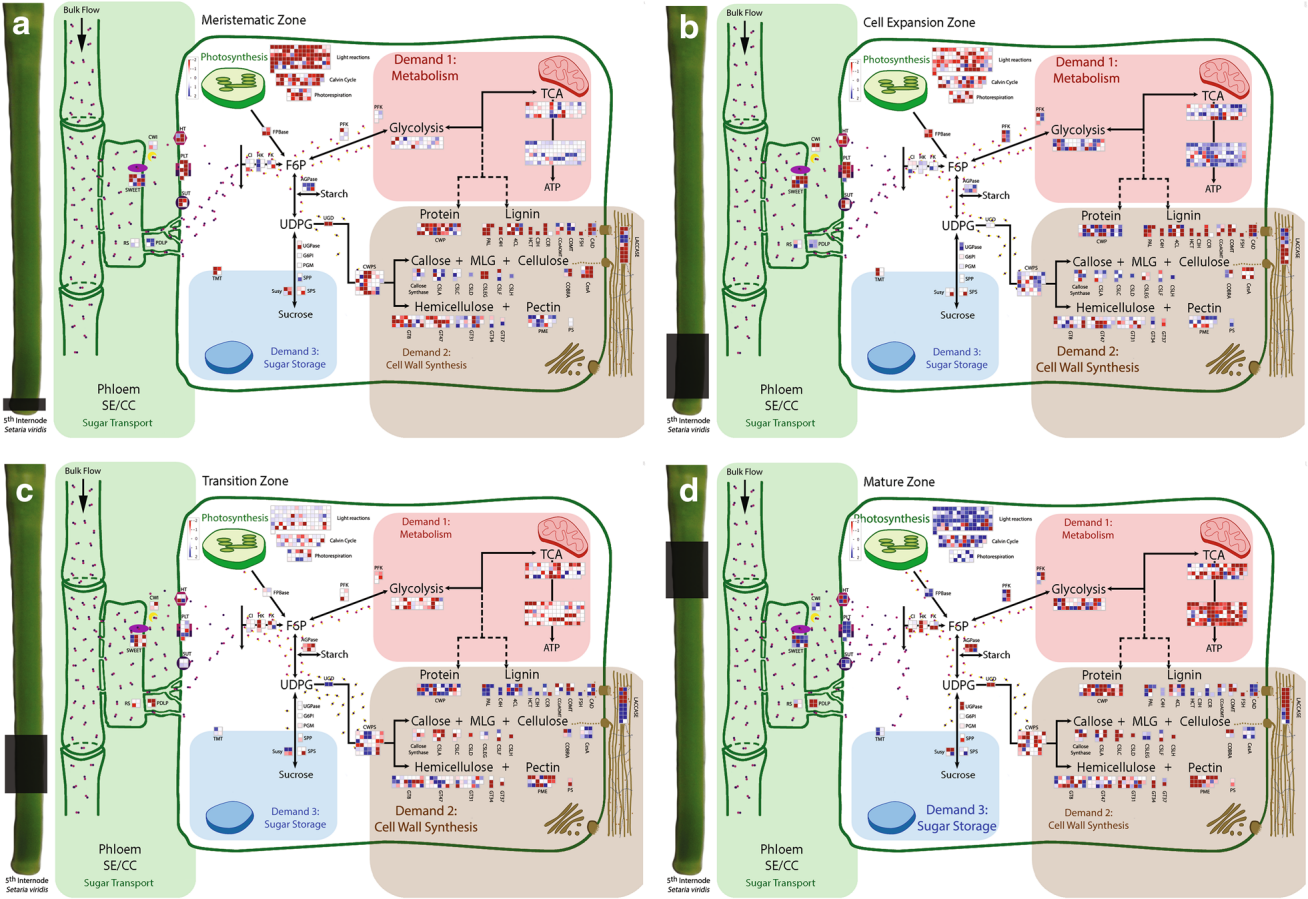
Schematic model of carbon demands during internode development based on transcriptomic analysis. A schematic pathway of carbon import into the developing *S. viridis* fifth internode, showing the three major demands on carbon supply. A Mapman pathway file (Additional file 4 and Additional file 5) was created to generate this visualisation of gene expression with regard to each carbon demand (also using the updated *S. viridis* mapping file, Additional file 3, and the experiment file, Additional file 2). Genes were assigned into categories (as labelled on the diagram) and their expression values were displayed as squared log_2_ fold change from the squared average of the four internode zones for each gene. Up-regulated genes are shown in *blue*, whilst down-regulated genes are in *red*. The.mp4 animation (Additional file 6) changes from meristematic zone (**a**), to cell expansion zone (**b**), to transition zone (**c**), to mature zone (**d**) indicating the internode region with a transparent *grey box* on the left and highlighting the greatest demand for carbon in each region. *MLG* mixed linkage glucans, *RS* raffinose synthase, *PDLP* plasmodesmata localised proteins, *SWEET* sugars will eventually be exported transporters, *CWI* cell wall invertase, *HT* hexose transporters, *PLT* polyol transporters, *SUT* sucrose transporters, *CI* cytosolic invertases, *HK* hexokinase, *FK* fructokinase, *FPBase* fructose-1,6-bisphosphatase, *F6P* fructose-6-phosphate, *PFK* phosphofructokinase, *AGPase* glucose-1-phosphate adenylyltransferase, *UGD* UDP-glucose 6-dehydrogenase, *UGPase* UDP-glucose phosphorylase, *G6PI* glucose-6-phosphate isomerase, *PGM* phosphoglucomutase, *SPP* sucrose phosphate phosphatase, *SPS* sucrose phosphate synthase, *Susy* sucrose synthase, *TMT* tonoplast membrane transporter, *CWPS* cell wall precursor synthesis, *CWP* cell wall protein, *PAL* phenylalanine ammonia-lyase, *C4H* cinnamate-4-hydroxylase, *4CL* 4-hydroxycinnamate CoA ligase, *HCT* hydroxycinnamoyl transferase, *C3H* coumarate 3-hydroxylase, *CCR* cinnamoyl-CoA reductase, *CCoAOMT* caffeoyl CoA 3-*O*-methyltransferase, *COMT* caffeic acid *O*-methyltransferase, *F5H* ferulate 5-hydroxylase, *CAD* cinnamyl alcohol dehydrogenase, *CSLA-H* cellulose synthase-like A-H, *CesA* cellulose synthase complex, *GT* glycosyltransferase, *PME* pectin methylesterase, *PS* pectin synthesis, *SE* sieve element, *CC* companion cell. *Blue* and *purple dots* are representative of sugar flow

To encapsulate the effectiveness of this experimental system for gene discovery and manipulation in a variety of fundamental biological processes, Mapman was used to visualise the expression of gene families relating to three major carbon demands of a developing grass culm (Fig. 6). In the meristematic zone, gene expression suggested predominantly symplasmic import of photoassimilate and utilisation of starch for cell metabolism and energy production (demand 1), since expression of sugar transporters, including *SWEET*s (sugar effluxers [29], *SUT*s (sucrose-H^+^ symporters [30]), *HT*s (hexose transporters [31]), *PLT*s (polyol transporters [32]), and *CWI* (cell wall invertase [33]) was low (Fig. 6). Gene expression in the cell expansion zone also suggested that photoassimilate is imported predominantly symplasmically [i.e. low expression of *SWEET*s, *SUT*s, *HT*s, *PLT*s, and *CWI*s and higher expression of plasmodesmatal located proteins (*PDLP*s)] not only in this zone and is directed towards demand 1 (energy/metabolism), but also primary cell wall synthesis, including hemicellulose, pectin, and cell wall protein synthesis (demand 2) (Fig. 6). The transitional zone is a pivotal developmental stage switching from predominantly metabolism (demand 1) and symplasmic photoassimilate import to a highly significant up-regulation of genes associated with apoplasmic sugar transport, coupled with a major switch and a re-direction of carbon flow to secondary cell wall synthesis (demand 2) (Fig. 6). The mature zone showed down-regulation of genes linked to metabolism (demand 1) and cell wall synthesis (demand 2), whilst apoplasmic transport genes (*SWEET*s, *SUT*s, *HT*s, *PLT*s, and *CWI*s) and vacuolar sugar storage (demand 3) genes (tonoplast monosaccharide transporters—*TMT*s [34]) showed further up-regulation. This suggested that the mature zone had ceased most of its transcriptional activity related to secondary cell wall synthesis and is actively transcribing genes that are associated with apoplasmic unloading of photoassimilate for storage.

## Discussion

*Setaria viridis* is becoming more widely accepted as a C_4_ grass model species, especially within the photosynthesis research community. Here we have presented the first transcriptomic analysis of this model system in tissue relevant to bioenergy research, at a gene discovery level. We have identified and shown that a developing *S. viridis* internode contains (i) an active meristematic zone that utilises starch and likely symplasmically imported sucrose and malate as a carbon source to drive metabolism, energy production, and synthesis of DNA, RNA, and protein, (ii) a zone of cell expansion with predominantly non-lignified primary cell walls which also utilises starch and symplasmically imported sugars, in the form of hexoses to produce energy, driving cell expansion, (iii) a transitional zone where cell expansion ceases, secondary cell wall synthesis is initiated and sugar import switches to a, likely, apoplasmic step, and (iv) a mature zone containing thickened, lignified cell walls, that is actively accumulating and storing carbon in the form of sucrose and malate via the expression of energy-dependent sugar transport genes, suggesting a, likely, apoplasmic step during the unloading of photoassimilate into this tissue.

Dye feeding experiments in sugarcane [35] and sorghum [36] have shown that 5(6)-carboxyfluorescein (CF) moves symplasmically from vascular phloem to storage parenchyma cells in mature stem sink tissues. Interestingly, high expression of energy-dependent sugar transport genes within mature internodes has also been observed, in sorghum [37]; however, the protein cellular localisation, and therefore precise role in sugar accumulation within grass stems has not yet been elucidated. The strong expression of *SWEET*, *SUT*, *HT*, *PLT*, and *CWI* genes in mature sink tissue of the *S. viridis* developing internode provides evidence that an apoplasmic step may exist in *Setaria*. Localisation and characterisation of these sugar transport proteins, however, will determine the cellular localisation of a possible apoplasmic unloading step, which may be vascular in origin or located on storage parenchyma cells to facilitate unloading of sugar into the apoplasm for storage and regulation of turgor. Whilst the role of these transporters in phloem unloading and assimilate storage in maturing internodes is yet to be investigated, this experimental system has provided gene candidates and a biological system for protein localisation and phenotyping of gene knockdown lines which will further unravel this biological process that is integral to controlling soluble sugar levels in C_4_ panicoid grasses.

Similarly, the clear transition from primary cell wall synthesis to secondary cell wall synthesis in this system, coupled with gene expression data, has uncovered numerous *S. viridis* gene candidates which can now be manipulated and studied under controlled conditions to further our understanding of cell wall biosynthesis in C_4_ grass stems. Cellulose synthase-like (CSL) families A, C, D, F, and H were all switched on in the meristematic and cell expansion zones of the internode, implicating a potential role in cell division and/or primary cell wall synthesis of hemicelluloses. As an example of gene discovery in this system, we will examine the proteins encoded by the CSLF and CSLH gene families. Homologues of CSLF and CSLH have been shown to produce mixed linkage glucans (MLGs) in primary cell walls of barley [38, 39] during wall expansion, and in secondary cell walls of rice stems [40]. The dataset presented here has identified a *CSLF* and a *CSLH* gene expressed in primary cell wall tissues, and a *CSLF* gene expressed in mature tissues (Fig. 6) differentiating transcriptional control over MLGs in primary and secondary cell walls of *S. viridis*. Characterisation in this biological system by protein localisation, promoter reporter constructs, and genetic knockdown using available *S. viridis* transformation techniques [12, 13] is now possible to further our understanding of the role of each of these genes in MLG deposition. Similarly, a number of gene isoforms can now be identified within this dataset for the manipulation and characterisation of lignin precursor synthesis, lignin polymerisation, cellulose synthesis, and hemicellulose synthesis, examining genes associated with both primary and secondary cell wall synthesis (Fig. 6). Expression of transcription factors in this experimental system was not characterised here but would also be of great interest when manipulating cell wall deposition and composition in this experimental C_4_ panicoid grass system.

## Conclusions

The developing internode characterised here is a valuable experimental system for gene discovery related to a variety of fundamental biological processes, especially for traits important in determining bioenergy crop yields. These include meristematic control of sink size, biosynthesis of cell walls, and accumulation of soluble sugars. Coupling this resource with genetic transformation techniques in *S. viridis* [12–14], generating controlled experimental data for the genetic manipulation of sink size, cell wall composition, and soluble sugar content in a C_4_ grass model system is now possible.

## Methods

### Plant growth conditions

*Setaria viridis* A10 was grown in 2 L of 7:3:5:5 top soil:sand:perlite:crushed clay with 1 g/L of Plantacote depot 4M© (14:9:15 NPK) inside a growth cabinet with 16/8 h, 28/20C, and 55/70 %rH day/nights. Plants were illuminated with ~600 μmol s^−1^ m^−2^ PAR from fluorescent lamps during light hours. After germination, plants were thinned to leave one plant per pot and were given 150 mL of water (with 1.5 mL/L of Previcur^®^ on the first watering) approximately 3 h into the light cycle every day.

### Identification of a developing internode

Plants were harvested at seven developmental stages. These were defined as the 6-leaf stage (emerging but still straight), 9-leaf (emerging but still straight), booting, half emerged head, anthesis (3/4 visible pollen), milky dough, and hard dough. Dry weights of whole plants and internodes were measured by freeze drying for ~5 days to determine biomass. Plant heights and internode lengths were also measured. Whole stems were frozen slowly at −20 °C and thawed before centrifuging through glass wool filters to obtain the soluble fraction of the stem. Sucrose was measured in the soluble fraction by FTIR spectroscopy as previously described [19].

### Histological determination of developmental zones within the internode

The fifth internode from the base of the main stalk harvested at the half emerged stage was photographed, dissected, and fixed in 4 % (*w/v*) paraformaldehyde/1.5 % (*w/v*) glutaraldehyde. Fixed tissue was halved longitudinally, embedded in 6 % agarose and sectioned longitudinally on a vibratome along the entire length of the internode at a thickness of 50 μm. These sections were stained with 10 μg/mL DAPI and viewed under UV illumination on a Zeiss Axiophot microscope (Carl Zeiss Pty. Ltd., Oberkochen, Germany, http://www.zeiss.com). Nuclei, and therefore cells, were counted along the length of the internode using the ITCN plugin for imageJ (http://imagej.nih.gov/ij/plugins/itcn.html). Nuclei density and cell size were calculated in a sliding window by dividing the number of each within the window by the width of the window at 5-μm interval. Cell size and nuclei density were immediately matched with defining features of the unsectioned half of the internode. Other internodes were quartered transversely, dehydrated through an ethanol series, and infiltrated with Technovit© (Heraeus Kulzer, Hanau, Germany, http://www.heraeus-kulzer.com). Technovit© blocks were polymerised and 4-μm transverse sections were cut on a microtome. Sections were stained with 0.05 % Toluidine blue and counterstained with Lugol’s iodine before viewing under brightfield illumination.

### Harvest of a developing internode

Eighty plants were harvested at the half emerged head stage (see “Results” section for details) from two crops grown under the same conditions (40 plants per crop) between 8 and 11 h into the light cycle. Harvest was staggered as plants reached the half emerged stage from 25 to 28 days after sowing (DAS). Plants were photographed; height and fresh weight were measured to ensure uniformity, and the fifth internode was dissected immediately after removal from the growth cabinet. The leaf sheath was removed from the fifth internode, and the intercalary meristem, cell expansion, transition, and mature zones were dissected and snap frozen in liquid N_2_. Since each dissected segment was only 5–15 mg, tissue was pooled from ten plants for each replicate to generate four biological replicate pools for each developmental zone.

### Acetyl bromide lignin

Twenty mg of fresh, ground tissue was weighed and washed sequentially with water, ethanol, and acetone before being freeze dried overnight. Lignin was solubilised by incubating in a thermomixer at 55 **°**C and 600 rpm for 150 min in 25 % (*v/v*) acetyl bromide prepared with glacial acetic acid. After cooling, acetic acid: 2 M NaOH mixture (50:9 *v/v*) and 0.5 M hydroxylamine chlorhydrate were added and the absorbance was read at 280 nm. Acetyl bromide lignin content was calculated using an extinction coefficient of 20.48 that was previously determined for corn stover [41].

### Primary metabolites

Soluble sugars and malate were measured enzymatically in ethanolic extracts from 20 mg fresh weight of frozen, ground tissue powder, and starch was measured enzymatically in the ethanol-insoluble residue, as previously described [42].

### RNA extraction, library preparation, and sequencing

Samples were ground to a powder at −60 °C using a cryogenic grinding robot (Labman Automation, Middlesborough, UK; http://www.labman.co.uk). RNA was extracted from aliquots (80–100 mg FW) of frozen tissue powder using a standard TRIzol© (Life technologies, Carlsbad, USA, http://www.lifetechnologies.com) extraction method and precipitated with EtOH. DNase treatment was performed using Turbo DNA-*free*™ kit (Life technologies, Carlsbad, USA, http://www.lifetechnologies.com) as per the manufacturer’s instruction. RNA quality was assessed by analysis with an RNA 6000 Nano kit and Bioanalyzer 2100 (Agilent Technologies; Santa Clara, CA, USA; http://www.agilent.com). RIN scores of above eight were considered acceptable. RNAseq libraries were generated with polyA enrichment and rRNA depletion using NEBNext^®^ Ultra™ Directional RNA Library Prep Kit (NEB, Ipswich, USA, http://www.neb.com) for Illumina^®^ and were sequenced on an Illumina^®^ HiSeq 2500 at the Max Planck Genome Centre, Cologne, Germany.

### RNAseq analysis

Illumina 100 bp paired-end reads from four biological replicates of the four internode zones were imported into CLC genomics workbench 8 (CLCbio, Aarhus, Denmark, http://www.clcbio.com) for adapter removal, trimming, mapping, and read counting. A single-end Illumina read library, generated from a mixture of leaf, stem, node, crown, root, spikelet, floret, and seed tissue across three developmental stages (seed germination, vegetative growth, and reproduction), was downloaded from the NCBI short read archive (SRA784127), imported into CLC genomics workbench 8, and analysed in parallel with the internode data. This ‘whole plant’ sample was used as an indication of background expression for the assessment of stem- and zone-specific gene expression.

Adapter sequences were trimmed from all reads. Low-quality reads were trimmed using a modified Mott trimming algorithm within CLC genomics 8. Reads with a quality score limit below 0.05 and regions containing more than two base ambiguities were trimmed. Reads were then mapped to the *S. italica* genome version 2.1 with gene annotations obtained from Phytozome 10.1 on the 29th April, 2015 [16], since an annotated *Setaria viridis* transcriptome and genome were not publically available. Mapping was performed using the principles of Mortazavi et al. [43] using a mismatch cost of 2, insertion cost of 3, deletion cost of 3, a length fraction of 0.8, and a similarity fraction of 0.8. Paired read distances were set at 180–660 bp, based on Bioanalyser runs of the RNA libraries, and the maximum number of hits for a read was set at 10. Fragments per kilobase per million (FPKM) was calculated for each gene, and EDGE test [44] and ANOVA false discovery rates were calculated. Log_2_ fold change between internode regions was calculated against the average of the four zones for each gene using x^2^ transformed data. Mapman bins were assigned to genes using the Mapman Phytozome v9.0 mapping file for *S. italica* downloaded on the 12th May, 2015. Genes encoding sugar transporters and cell wall synthesis related enzymes/proteins were manually annotated by BLAST against maize, rice, and sorghum proteome sequences and Mapman bins were assigned accordingly. A file containing FPKM, log_2_ fold change, Mapman bin numbers, and gene annotations can be found in Additional file 2. This file was used, along with our updated Mapman mapping file (Additional file 3) to generate Mapman visualisations of the RNAseq dataset.

Principal component analysis was performed in ‘The Unscrambler^®^ X 10.2’ (CAMO) with standard deviation-weighted FPKM values using the NIPALS algorithm and full cross validation [45]. Venn diagrams were generated using ‘Venny 2.0’ [46]. Gene categories of interest were selected using the Mapman binning system in Additional file 3 and average FPKM was calculated for each category.

## Additional files

10.1186/s13068-016-0457-6 RNAseq mapping statistics. Number of reads, reads after trimming, mapped reads (%) and unique reads (%) for each RNAseq sample described in the manuscript.
10.1186/s13068-016-0457-6 RNAseq data. A spreadsheet containing FPKM, log_2_ fold change, Mapman bin numbers, and gene annotations for the RNAseq data generated for this manuscript.
10.1186/s13068-016-0457-6 Updated Mapman mapping file. A mapping file that can be imported into Mapman containing some manual annotations that were used for this manuscript.
10.1186/s13068-016-0457-6 Mapman pathway image. A schematic overview of the biological processes occurring in a developing *S. viridis* internode that can be imported into Mapman to map genes and metabolite levels onto this image.
10.1186/s13068-016-0457-6 Mapman pathway file. A Mapman pathway file that maps relevant genes onto the image in Additional file 4.
10.1186/s13068-016-0457-6 Schematic model of carbon demands during internode development based on transcriptomic analysis. Figure 6 from the manuscript compiled as an .mp4 video.

## Abbreviations

DAPI: 4′,6-diamidino-2-phenylindole
MsZ: meristematic zone
CEZ: cell expansion zone
TZ: transition zone
MZ: mature zone
PCA: principle component analysis
FPKM: fragments per kilobase per million reads
SWEET: sugar efflux proteins
SUT: sucrose transporters
HT: hexose transporters
PLT: polyol transporters
CWI: cell wall invertase
TMT: tonoplast monosaccharide transporters
PDLP: plasmodesmatal localised proteins
CSL: cellulose synthase-like proteins
