# Supplementary figures and images for: A developing Setaria viridis internode: an experimental system for the study of biomass generation in a C4 model species
Source: Biotechnol Biofuels. 2016 Feb 24;9:45. doi: 10.1186/s13068-016-0457-6 (PMC4766645; doi:10.1186/s13068-016-0457-6)

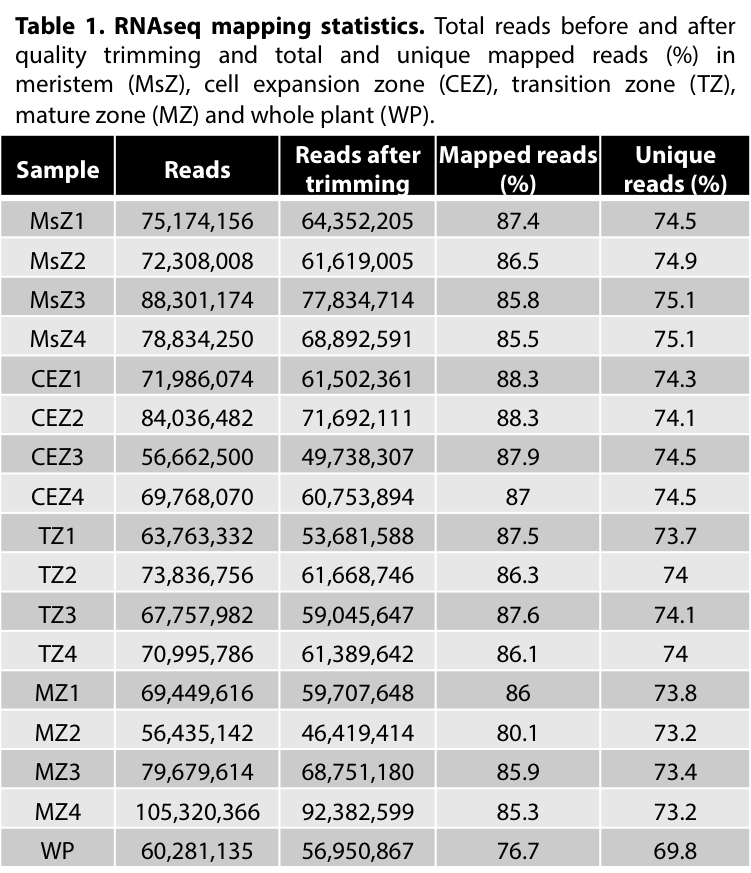

Supplement: Supplementary file 1 — 10.1186/s13068-016-0457-6 RNAseq mapping statistics. Number of reads, reads after trimming, mapped reads (%) and unique reads (%) for each RNAseq sample described in the manuscript. [file 13068_2016_457_MOESM1_ESM.png]

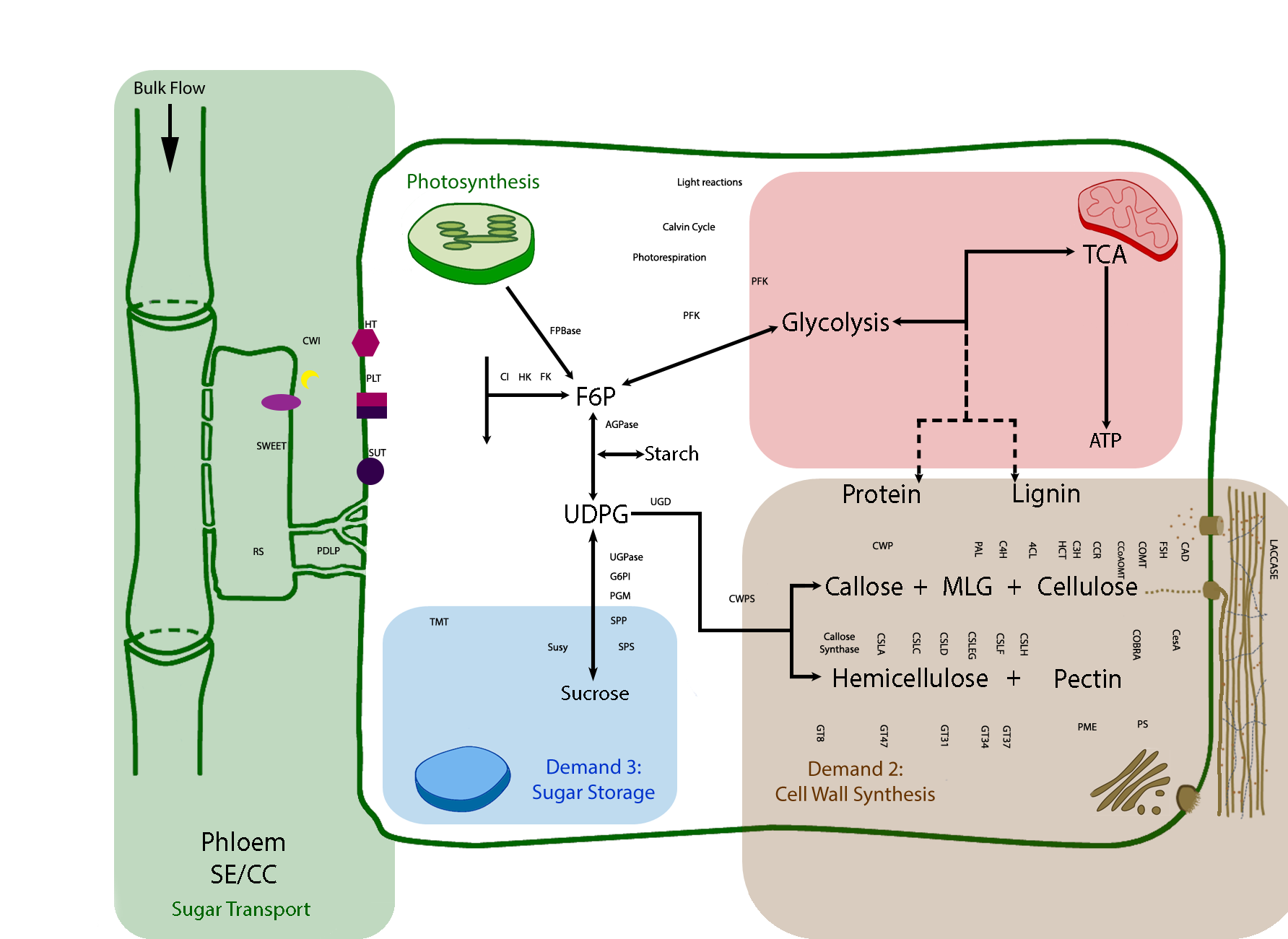

Supplement: Supplementary file 4 — 10.1186/s13068-016-0457-6 Mapman pathway image. A schematic overview of the biological processes occurring in a developing S. viridis internode that can be imported into Mapman to map genes and metabolite levels onto this image. [file 13068_2016_457_MOESM4_ESM.png]
